# Assessing the efficacy of a modified assertive community-based treatment programme in a developing country

**DOI:** 10.1186/1471-244X-10-73

**Published:** 2010-09-15

**Authors:** Ulla A Botha, Liezl Koen, John A Joska, Linda M Hering, Piet P Oosthuizen

**Affiliations:** 1 Department of Psychiatry, University of Stellenbosch, Tygerberg, South Africa; 2 Department of Psychiatry, University of Cape Town, Cape Town, South Africa; 3 Associated Psychiatric Hospitals, Cape Town, South Africa

## Abstract

**Background:**

A number of recently published randomized controlled trials conducted in developed countries have reported no advantage for assertive interventions over standard care models. One possible explanation could be that so-called "standard care" has become more comprehensive in recent years, incorporating some of the salient aspects of assertive models in its modus operandi. Our study represents the first randomised controlled trial assessing the effect of a modified assertive treatment service on readmission rates and other measures of outcome in a developing country.

**Methods:**

High frequency service users were randomized into an intervention (n = 34) and a control (n = 26) group. The control group received standard community care and the active group an assertive intervention based on a modified version of the international model of assertive community treatment. Study visits were conducted at baseline and 12 months with demographic and illness information collected at visit 1 and readmission rates documented at study end. Symptomatology and functioning were measured at both visits using the PANSS, CDSS, ESRS, WHO-QOL and SOFAS.

**Results:**

At 12 month follow-up subjects receiving the assertive intervention had significantly lower total PANSS (p = 0.02) as well as positive (p < 0.01) and general psychopathology (p = 0.01) subscales' scores. The mean SOFAS score was also significantly higher (p = 0.02) and the mean number of psychiatric admissions significantly lower (p < 0.01) in the intervention group.

**Conclusions:**

Our results indicate that assertive interventions in a developing setting where standard community mental services are often under resourced can produce significant outcomes. Furthermore, these interventions need not be as expensive and comprehensive as international, first-world models in order to reduce inpatient days, improve psychopathology and overall levels of functioning in patients with severe mental illness.

## Background

In recent years there has been a worldwide focus on assertive community interventions in an attempt to address some of the repercussions of the implementation of deinstitutionalization [1-6]. Although these interventions have often been implemented under different names such as assertive outreach, intensive case management and assertive community treatment, essentially they have had the same core characteristics [4,7] (See Additional file 1)

A Cochrane review published in 1998 concluded that assertive interventions exhibited several advantages above standard care, such as improved engagement with services, reduction in readmissions and days spent in hospital (DIH), benefits in employment and accommodation status, as well as improved patient satisfaction [3]. The review found no differences in severity of psychopathology or level of functioning, but reported a reduction in inpatient costs, even though no benefits were shown when other costs were taken in account.

With the exception of Lambert et al, recent publications have failed to replicate the previously reported efficacy of assertive interventions over standard care models, with a number of randomized controlled trials showing no advantage for Assertive Community Treatment (ACT) in reducing inpatient care and other clinical outcomes [2,4-6,8,9]. Improved engagement with services and increased patient satisfaction has been the only consistently positive findings. One of the explanations offered is the likelihood that so-called "standard care" has become more comprehensive in recent years, incorporating some of the salient aspects of assertive models in its modus operandi [2,5,10]. Some studies were criticized for not defining control groups well enough, since "treatment as usual" may differ between settings and should therefore be properly defined as a separate intervention [10]. Another possible explanation is the fact that hospital readmissions have been the most frequently measured and often primary outcome. This variable may be particularly difficult to reduce in a system where recidivists only have access to beds when they are extremely ill and are again discharged before they are completely stable [2,5].

Psychiatric services in some developing countries have had similar experiences to those of developed countries with regards to demand for in-patient services and recidivism [1,11-13]. The impact of deinstitutionalization became evident only in retrospect, and has placed a significant burden on already overburdened community services [12,14]. Community psychiatric services in South Africa are based in primary health care institutions and have to contend with a lack of resources, particularly services offering residential specialized care. In many cases these services still rely heavily on resources that are only accessible through hospital-based care. High rates of unemployment, poor social circumstances, substance abuse and high levels of violence and crime, further contribute to the unique challenge mental health services face in developing countries.

In a previous paper from our group, we found the characteristics of high frequency users (HFUs) in the South African setting to be quite similar in profile to those described in the international literature [15]. The paucity of resources was shown to be amongst the driving forces behind high frequency use, along with poor medication adherence and substance abuse. Stein et al suggested that South African clinicians should develop their own model of providing community care through strengthening of existing community structures and stressed that intensive care with small caseloads, may not be realistic in the South African setting [16].

It is against this backdrop that the state psychiatric management team in the Western Cape Province, South Africa, introduced an assertive community treatment program for each of the three regional psychiatric hospitals in an attempt to reduce demand for inpatient beds and to alleviate some of the pressure on community psychiatric services [1]. Since the model of care provided by such teams in high income countries would not be realistic or cost-effective in the South African setting, the international model was modified to allow for larger caseloads and consequently less frequent contacts. See table 1 for comparisons between ACT teams and standard community mental health teams.

**Table 1:** Work style of ACT team compared to standard care

	**ACT team**	**Community Mental Health team**
Overall patient load	80-100 patients	± 600 patients excluding assessments of new patients
Individual caseload	Maximum 35	250
Workstyle	Key workers act as care coordinator bur caseloads are shared	Individual caseloads
Site of most visits	>50% contacts are home visits	Office based
Engagement	Assertive; focus on engagement.	Non-assertive, no follow-up of missed appointments/reports of non-compliance
Working hours	Office hours	Office hours
24 hour cover	Patients referred to hospital-based after-hours service coordinated by ACT.	After-hours service of catchment area.
Frequency of contacts	Individualized according to patient need; fortnightly	Depends on caseloads, varies between monthly to three monthly.
Disciplines available	Full-time psychiatrist, social worker, psychiatric nurse, access to psychologist, occupational therapist, dual diagnosis outpatient service.	Full-time psychiatric nurse, access to social worker and psychiatrist, varied access to occupational therapist and psychologist.

## Aim

The purpose of this study was to determine the impact of a tailored, assertive treatment service on readmission rates and other measures of outcome in HFUs of psychiatric services in a developing country

## Methods

This study was conducted at Stikland Hospital, one of the three large state psychiatric hospitals in Cape Town, South Africa. The hospital, along with two others, provides inpatient services to the whole of the Western Cape Province, servicing a population of approximately 5 million people. The combined in-patient numbers for patients with severe mental illness in the three hospitals is approximately 450. The Stikland Hospital ACT Team consisted of a full-time psychiatrist, a social worker and a chief professional nurse.

All clients who presented for admission to Stikland Hospital over a pre-defined period in 2007/08 and who had a previously established, documented diagnosis of schizophrenia or schizo-affective disorder (DSM-IV-TR), were considered for inclusion [17]. In order to be included, participants had to give written, informed consent. The study was approved by the research ethics committees of both the Universities of Stellenbosch and Cape Town. The research study was conducted parallel to a service component into which patients not meeting research criteria, but with a similar pattern of high frequency use, were recruited. Research numbers therefore do not reflect overall caseloads; patients participating in the research constituted only one third of the overall caseload. Originally, the research project was intended as a multi-site project, covering the three catchment areas in the metro, but due to high turnover in staff, the study could not be completed at the other two institutions. This reduced the number of participants who were included in the study, but had the advantage that a single investigator (UB) performed all the assessments.

To be included as HFUs participants had to fulfill the full criteria as described in Additional file 2: Table S1. We utilized a modified version of Weiden's HFU-criteria adapted to local circumstances [18] (Additional file 2: Table S1). Participants were excluded if they had (1) a severe, unstable, co-morbid, medical illness (2) were unable to give written informed consent or (3) if another co-morbid Axis I or II diagnosis, other than schizophrenia or schizo-affective disorder, was the current focus of treatment.

**Figure 1:**
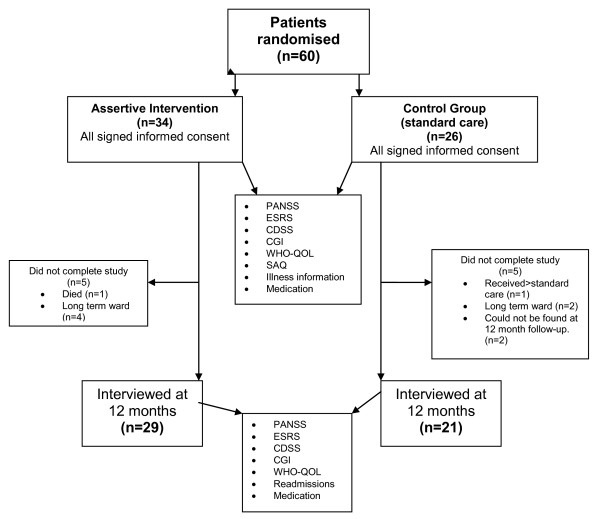
**Study methodology**. 60 participants identified as HFUs who provided informed consent, were randomized using standardized tables. 34 participants were randomised to the intervention group and 26 to the treatment as usual group. Participants from both groups were assessed at inclusion and rating scales as described in the methods section were performed at each of these visits. Participants from both groups were assessed again after 12 months for the final assessment. On this visit data was collected and rating scales were performed again. In each group, 5 participants did not complete the study.

After inclusion, 60 participants identified as HFUs who provided informed, written consent, were randomized using standardized tables to either the intervention group or the treatment as usual group (see Figure 1).

Participants from both groups were assessed at inclusion, prior to discharge and at 12 months after inclusion. All assessments were done by a single investigator, and all data was entered into an electronic Case Report Form (eCRF). At each of these visits, the following information was gathered and rating scales administered:

○ Positive and Negative Symptom Scale (PANSS) [19]

○ Extrapyramidal symptom rating scale (ESRS) [20]

○ Calgary Depression Scale for Schizophrenia (CDSS) [21]

○ Social and Occupational Functioning Assessment Scale (SOFAS) [22]

○ World Health Organization Quality of Life questionnaire (WHO-QOL) [23]

○ Information about diagnosis, illness and medication (obtained from medical folder)

○ Confirmation of demographics and living arrangements

Participants from the treatment as usual group were discharged into the existing community mental health service and were only contacted again after 12 months for the final assessment. Participants from the intervention group were each assigned a key worker in the form of a senior social worker or a chief professional nurse. Key workers started engaging subjects and carers prior to discharge, with the primary focus on building a therapeutic relationship.

The nature of the intervention was tailored as closely as possible to the international model of assertive community treatment, with the two main exceptions being the size of caseloads and frequency of visits. It was agreed at the outset that caseloads carried by international teams would not be realistic in the context of an under-resourced, developing country. (See table 1 for characteristics of team.) A consensus caseload number of 80 patients per team was reached, with individual caseloads not exceeding 35. Fidelity to the international model was assessed with the Dartmouth Assertive Community Treatment Scale (DACTS) with a total score of 3.1, indicating moderate fidelity[24]. The DACTS was developed by Teague et al as an independent scale used to assess adherence to evidence-based practices particular to assertive community treatment. The scale contains 28 program-specific items, wherein each item on the scale is rated on a 5-point scale ranging from 1 to 5 indicating the degree to which principles were implemented. The scale is accompanied by a guideline for scoring of each item. Higher scores (4-5) are indicative of high fidelity, with scores between 3 and 4 indicating moderate fidelity and those below 3, low fidelity[24].

Key workers acted as main care coordinators, but caseloads were often shared between members of the team. A major focus of the team was on engagement and maintenance of adherence to treatment. Since resources were limited, the team focused on strengthening access to existing resources in the community and building new ties with organizations that may offer additional services. Patients were frequently referred to occupational therapy and psychology services, although no full time staffing was available from these disciplines. Since there are no inpatient dual diagnosis rehabilitation facilities in the area, patients were referred to mainstream programs when this service was required. The majority of contacts (>50%) were in the community, mainly in the form of home visits. The team was based at Stikland Hospital. This held both advantages and disadvantages. On the one hand, the team was able to draw from the various resources in the hospital setting to strengthen the service it provided, such as access to day centres, occupational therapy assessments and coordination of medication issuing. One major disadvantage of the teams' location was the historical, custodial reputation of state institutions. The team therefore had to work harder to challenge misconceptions about its purpose.

At 12 month follow-up, information was collected about readmissions and changes in medication. Remission rates were based on Andreasens' criteria [25]. Patients in the intervention group remained in the service and those in the control group were, at study end, given the option to be included in the service as well. There was no official drop-out policy and none of the intervention patients dropped out during the course of the study.

### Statistical Analysis

All data were entered into a single, electronic database. Statistical Analysis was done with Statistica version 9 software (Statsoft, Inc 2009). As some of the data was descriptive in nature, results are provided as means with standard deviations, where appropriate. Categorical variables were compared using chi-square or Fisher's exact test, where applicable. Differences in groups in terms of continuous variables were analyzed using Student's T-test. All statistical tests were two-sided and a significance level of 0.05 was used throughout.

## Results

A total of 34 participants were included in the intervention arm. Five of these did not complete the study: three were never discharged during the study period and one died before study completion. The other was re-admitted within two weeks after discharge and then transferred to a long stay ward where he remained until study completion. No data was therefore included for the first three but for the last two data from study visit 1 as well as the period they remained on the study was included. Of the 26 participants who initially consented to act as controls, 21 completed the study. Two could not be traced after 12 months, one had been seen monthly by a psychiatrist through-out the year and was therefore considered not to have received standard care. The other two were transferred to long-stay wards shortly after inclusion. Almost two thirds of patients in both groups were male and approximately the same number was unmarried. With one exception from both groups, all patients were unemployed and lived in their family home. Twenty-three intervention and nineteen control participants received disability grants. See Table 2 for detailed demographics.

**Table 2:** Demographic differences between Intervention group and Control Group

		Intervention			Control						
		**Mean (±SD)**	**n**	**%**	**Mean (±SD)**	**n**	**%**	**x**^2^	**t-value**	**df**	**p**

**Age**		30.55 (±9.09)	31		34.81 (±11.02)	21			-1.52	50	0.13

**Gender**	male		23	74.19		15	71.43	0.05		1	0.83
					
	female		8	25.80		6	28.57				

	mixed*		29	93.55		19	90.48				
					
**Ethnicity**	black*		1	3.23		2	9.52	1.55		2	0.46
					
	caucasian		1	3.23		0	0				

**Residential area**	metro**		31	100		19	90.48				0.19
					
	rural		0	0		2	9.52				

**Education level**	elementary		16	51.61		7	33.33				
					
	secondary		12	38.71		10	47.62	2.89		3	0.41
					
	≥Gr12		3	9.68		3	14.29				
					
	none		0	0		1	47.62				

**Marital status**	single		25	80.65		16	76.19				
					
	married		4	12.90		2	9.52	0.95	2		0.62
					
	divorced		2	6.45		3	14.29				

**Employment Status**	unemployed		30	96.77		21	100	0.69		1	0.41
					
	casual***		1	3.23		0	0				

**Disability grant**	yes		23	74.19		19	90.48	2.14		1	0.14
					
	no		8	25.80		2	9.52				

*Mixed refers to participant with mixed African-Caucasian ancestry. Black refers to black African participants.

**Participants who live within the city limits of the City of Cape Town

***Participants who are employed on a part-time basis. Note that no participants were fully employed

Baseline scores in psychopathology were similar between the groups, except for a significantly higher mean score on the PANSS Negative Scale for the intervention group (p = 0.01). At 12 month follow-up, the intervention group had significantly lower scores in the subscales for PANSS positive (p < 0.01) and general psychopathology symptom scales (p = 0.01), as well as for PANSS total scores (p = 0.02). Also, the difference in PANSS Negative Scores was no longer significant. The mean SOFAS score was significantly higher in the intervention group (p = 0.02). No significant differences were found in scores for CDSS and WHO-QOL. There was no significant difference in the use of depot medication, nor was there any significant difference in ESRS scores. Although there was a large numerical difference in the number of participants who reached remission between the two groups, this number did not reach significance. The risk for readmission was significantly higher in the control group with 10 patients (n = 31) in the intervention group being readmitted during the course of the year and 15 in the control group (n = 21). The mean number of admissions per capita for the intervention group was 0.41 and 1.19 in the control group. (p < 0.01) The mean number of inpatient days was also significantly higher in the control group, both for psychiatric (p = 0.02) and non-psychiatric admissions (p = 0.04). (see Table 3 &4 for full results).

**Table 3:** Differences in clinical outcomes between Intervention group and Control Group (1)

Item		Intervention		Control				
		
		Mean (±SD)	n	Mean (±SD)	n	t-value	df	p
**Baseline (mean)**	PANNS-P total	32.29 (±5.62)	31	31.43 (±5.21)	21	0.56	50	0.58
	
	PANNS-N total	25.06 (±6.82)	31	20.00 (±6.80)	21	2.63	50	0.01*
	
	PANNS-G total	48.16 (±9.21)	31	45.67 (±6.37)	21	1.08	50	0.29
	
Intervention (n = 31) Control (n = 21)	PANNS-Total	105.52 (±18.58)	31	97.10 (±15.20)	21	1.72	50	0.09
	
	*SOFAS*	34.29 (±3.58)	31	36.29 (±6.37)	21	-0.89	50	0.38
	
	CDSS	2.35 (±18.58)	31	1.05 (±1.47)	21	1.58	50	0.12
	
	ESRS-questionnaire	3.16 (±2.48)	31	2.43 (±2.40)	21	1.06	50	0.29
	
	ESRS-parkinsonism	8.84 (±7.28)	31	8.81 (±5.55)	21	0.02	50	0.99
	
	ESRS-dystonia	0.00 (±0.00)	31	0.10 (±0.44)	21	-1.22	50	0.23
	
	ESRS-dyskinetic	0.61 (±2.38)	31	0.57 (±2.62)	21	0.06	50	0.95

**Endpoint**	PANNS-P total	12.52 (±6.0)	29	19.38 (±8.8)	21	-3.28	48	0.00*
	
	PANNS-N total	16.55 (±6.1)	29	19.33 (±4.6)	21	-1.76	48	0.09
	
	PANNS-G total	28.45 (±8.2)	29	34.81 (±9.1)	21	-2.58	48	0.01*
	
	PANNS-Total	57.52 (±17.4)	29	73.52 (±19.2)	21	-3.07	48	0.00*
	
Intervention ( n = 29) Control (n = 21)	SOFAS	61.97 (±9.1)	29	54.90 (±10.8)	21	2.50	48	0.02*
	
	CDSS total	0.69 (±1.4)	29	0.81 (±3.3)	21		48	0.86
	
	ESRS-questionnaire	1.90 (±1.23)	29	1.90 (±1.51)	21	-0.02	48	0.98
	
	ESRS-parkinsonism	9.03 (±8.20)	29	0.48 (±8.07)	21	0.48	48	0.63
	
	ESRS-dystonia	0.00 (±0.00)	29	0.00 (±0.00)	21		48	
	
	ESRS-dyskinetic	0.55 (±1.24)	29	0.57 (±1.57)	21	-0.05	48	0.96

*Significance at p < 0.05

**Table 4:** Differences in clinical outcomes between Intervention group and Control Group (2)

Item		Intervention			Control						
	
		Mean (±SD)	n	%	Mean (±SD)	n	%	**x**^2^	t-value	df	p-value
**Remission**	yes		13	44.83		6	28.57	1.367		1	0.24
					
	no		16	55.17		15	71.43	7			

**Readmission**	yes		10	34.48		15	71.43	6.65		1	0.01*
					
	no		19	65.52		6	28.57				
	
	number readmissions	0.41 (±0.63)	29		1.19 (±0.98)	21			3,41	48	0.00*
	
	days in hospital (DIH)	24.69 (±47.43)	29		67.19 (±76.31)	21			-2.43	48	0.02*
	
	non-psychiatric DIH	0.07 (±0.37)	29		2.33 (±5.65)	21			2.16	48	0.04*

*Significance at p < 0.05

## Discussion

We report on the first detailed prospective study of assertive community treatment in South Africa. Our results suggest that assertive community treatment may not only reduce readmission rates in a setting with limited resources, but may also impact on the severity of psychopathology and level of functioning [2,4-6,8]. These findings appear to stand in contrast to those reported on by others in high income countries. Even though our team did not have a high fidelity as demonstrated by the DACT score, the service offered appeared to be significantly more effective than standard care in reducing readmissions and improving clinical outcomes.

The impact of assertive community treatment is likely to reside in the additional resources provided by the intervention in a poorly resourced setting. Existing community services are over-burdened with a rapidly growing population of mental health care users. Community mental health service are hampered by staffing shortages, limited access to residential care, restricted availability of vocational rehabilitation and related service. The high demand for services is fuelled by high rates of substance abuse, the HIV epidemic, and poor social conditions. The literature on ACT indicates that assertive interventions may be more effective where community services are less comprehensive [10,26]. Ironically, it is in these exact settings, often in developing countries, where assertive interventions may not be affordable or feasible.

Additional reasons for the positive outcome in this study include factors related to the establishment of a novel service. Sytema et al commented on the influence a newly established team may have on outcomes of a trial [5]. On the one hand, enthusiasm and motivation may be higher in a newly established team that has something to prove. On the other hand, there is the pressure of developing a new service that has never been tried before, especially in this case where the model of care has been adapted.

The positive effect of assertive community treatment in our setting is unlikely to be related to medication use and dose, since no significant differences were found between the two groups in this respect. Comparisons drawn between low frequency users (LFUs) and HFUs in the same population in the past, have shown a higher incidence in the use of depot medication in LFUs, which may improve overall compliance and prolong periods between admissions [15]. However, there was no difference in the use of depot medication between the two groups in this study at endpoint.

In addition to reduced admission rates, we also noted that participants in the intervention spent less time in hospital (referred to as days in hospital- DIH). Since the patients in the intervention group had more frequent service contacts, it is likely that intervention occurred earlier in the course of relapse and that patients from this group were therefore less severely ill on readmission than the patients in the control group. Also, patients in the intervention group had streamlined access to emergency and inpatient services, because of the involvement of the ACT team. The higher number of non-psychiatric inpatient days in the control group is probably a result of the pathways followed to admission. Due to the fact that there are limited bed vacancies at state institutions on the day admission is required, patients are often admitted to medical beds in secondary hospitals and put on a waiting list until beds become available at a psychiatric hospital. Patients in the ACT service did not follow this route, as one of the advantages of the service is the streamlined access to beds when in crisis.

One may speculate that these outcomes reflect more on the level of standard care in South Africa rather than the efficacy of the intervention offered. Also, some may question whether such a comparatively expensive intervention is an appropriate way to utilize the limited resources in developing countries. It is therefore reassuring and important to note that even with the modified caseloads and reduced frequency in contacts, significant outcomes can be produced on more than one level. This could be an indication that there may be a place for assertive community treatment strategies in developing countries, although these should be tailored to the needs and resources of the particular population and country. Therefore, with the clinical benefits of this particular intervention already demonstrated in our setting, we believe the next logical step should be an urgent cost-benefit analysis in order to present policy makers with the data needed to support funding for a wider roll-out of this program.

## Conclusion

This is the first study of its kind conducted in a developing country. The results indicate that assertive interventions in this setting need not consume resources to the degree that high income country models use to produce positive outcomes. Modified assertive interventions that focus on maintaining adherence and offering additional support may not only reduce inpatient days but also improve psychopathology in patients with severe mental illness. Standard community mental health services in developing countries often lack necessary resources and funding to provide comprehensive care to the severely ill, HFU patient. Ways should be explored in which traditional assertive models of care can be adapted within the financial constraints of limited budgets, while still retaining the core features necessary to bring about change.

## Limitations

This was an unblinded study, with all the inherent risks involved when this kind of methodology is used. Due to high staff turnover at other sites, numbers of subjects recruited were lower than expected, and from a single site only which could limit generalizability. The ethnic distribution in this sample is not representative of the entire population of the country, since the study was conducted in an area where the predominant ethnic representation is that of mixed race. Ideally, outcomes should be measured for longer than 12 months since some clinical outcomes may change over time.

## Declaration of competing interests

The authors declare that they have no competing interests.

## Authors' contributions

All authors conceived of and designed the study. UB acquired the data. PO performed the statistical analysis. UB prepared the first draft of the manuscript and PO and LK made significant contributions to the final draft. All authors read and approved the final manuscript.

## Pre-publication history

The pre-publication history for this paper can be accessed here:

http://www.biomedcentral.com/1471-244X/10/73/prepub

## Supplementary Material

Additional file 1 **Key Elements of ACT**. Contains description of core elements defining Assertive Community Treatment as defined by Burns et al. This model were adapteded from the original PACT model described by Stein and Test in 1992. Click here for file

Additional file 2 **Table S1**. Modified Weiden's criteria for differentiating high frequency (HFU) and low frequency (LFU) schizophrenia-spectrum disorder users of psychiatric services. Click here for file
