# Supplementary material for: Assessing the efficacy of a modified assertive community-based treatment programme in a developing country
Source: BMC Psychiatry. 2010 Sep 15;10:73. doi: 10.1186/1471-244X-10-73 (PMC2945974; doi:10.1186/1471-244X-10-73)
Supplement: Additional file 1 — Key Elements of ACT. Contains description of core elements defining Assertive Community Treatment as defined by Burns et al. This model were adapteded from the original PACT model described by Stein and Test in 1992. [file 1471-244X-10-73-S1.DOC]

**Key Elements of ACT model [7]**

| A core service team provides bulk of clinical care. |
| --- |
| Primary goal is improvement in patients' functioning. |
| Patient is assisted directly in symptom management. |
| Ratio of staff to patient should be small (no greater than 10-15:1). |
| Each patient is assigned a key worker responsible for comprehensive care. |
| Treatment is individualized between patients and over time. |
| Patients are engaged and followed up over time. |
| Treatment is provided in community settings. |
| Care is continuous over time and across functional areas. |
