# Supplementary material for: Assessing the efficacy of a modified assertive community-based treatment programme in a developing country
Source: BMC Psychiatry. 2010 Sep 15;10:73. doi: 10.1186/1471-244X-10-73 (PMC2945974; doi:10.1186/1471-244X-10-73)
Supplement: Additional file 2 — Table S1. Modified Weiden's criteria for differentiating high frequency (HFU) and low frequency (LFU) schizophrenia-spectrum disorder users of psychiatric services. [file 1471-244X-10-73-S2.DOC]

**Table S1**

**Modified Weiden's criteria for differentiating high frequency (HFU) and low frequency (LFU) schizophrenia-spectrum disorder users of psychiatric services**

| **General criteria** |
| --- |
| 1.)Schizophrenia or Schizo-affective Disorder |
| 2) Age 18-59 years (extremes included) |
| 3) Needs current treatment with antipsychotic |
| **Must meet General Criteria PLUS either (A) or (B) or (C) to be included** |
| (A) ≥3 admissions in 18 months/≥ 5 in 36 months |
| (B) ≥2 admissions in 12 months AND treated with clozapine |
| (C) ≥2 admissions in 12 months AND ≥120 days in hospital |

- **HFUs had to fulfill General Criteria PLUS one of A; B or**
